# Effectiveness of peer educators on the uptake of mobile X-ray tuberculosis screening at homeless hostels: a cluster randomised controlled trial

**DOI:** 10.1136/bmjopen-2015-008050

**Published:** 2015-09-21

**Authors:** Robert W Aldridge, Andrew C Hayward, Sara Hemming, Lucia Possas, Gloria Ferenando, Elizabeth Garber, Marc Lipman, Timothy D McHugh, Alistair Story

**Affiliations:** 1 Institute of Health Informatics, University College London, London, UK; 2 The Farr Institute of Health Informatics Research; 3 Royal Free London NHS Foundation Trust, London, UK; 4 Centre for Clinical Microbiology, Division of Medicine, University College London, London, UK; 5 Division of Infection and Immunity, Centre for Clinical Microbiology, University College London, London, UK; 6 University College London Hospitals, London, UK

## Abstract

**Trial design:**

Cluster randomised controlled trial.

**Objective:**

To compare current practice for encouraging homeless people to be screened for tuberculosis on a mobile digital X-ray unit in London, UK, with the additional use of volunteer peer educators who have direct experience of tuberculosis, homelessness or both.

**Participants:**

46 hostels took part in the study, with a total of 2342 residents eligible for screening. The study took place between February 2012 and October 2013 at homeless hostels in London, UK.

**Intervention:**

At intervention sites, volunteer peer educators agreed to a work plan that involved moving around the hostel in conjunction with the hostel staff, and speaking to residents in order to encourage them to attend the screening.

**Randomisation:**

Cluster randomisation (by hostel) was performed using an internet-based service to ensure allocation concealment, with minimisation by hostel size and historical screening uptake.

**Blinding:**

Only the study statistician was blinded to the allocation of intervention or control arms.

**Primary outcome:**

The primary outcome was the number of eligible clients at a hostel venue screened for active pulmonary tuberculosis by the mobile X-ray unit.

**Results:**

A total of 59 hostels were considered for eligibility and 46 were randomised. Control sites had a total of 1192 residents, with a median uptake of 45% (IQR 33–55). Intervention sites had 1150 eligible residents with a median uptake of 40% (IQR 25–61). Using Poisson regression to account for the clustered study design, hostel size and historical screening levels, there was no evidence that peer educators increased uptake (adjusted risk ratio 0.98; 95% CIs 0.80 to 1.20). The study team noted no adverse events.

**Conclusions:**

This study found no evidence that volunteer peer educators increased client uptake of mobile X-ray unit screening for tuberculosis. Further qualitative work should be undertaken to explore the possible ancillary benefits to peer volunteers.

**Trial registration number:**

ISRCTN17270334.

**Strengths and limitations of this study**

- Our study attempts to compare current practice used to encourage homeless people to be screened for tuberculosis, with the additional use of volunteer peer educators who have direct experience of tuberculosis, homelessness or both, using a rigorous cluster randomised controlled design.
- Our study has high levels of internal validity and used a pragmatic study design to analyse a challenging population and setting.
- Particular strengths of the study design include allocation concealment during randomisation and blinded statistical analysis of data by intention-to-treat.
- A limitation is that the study was not powered to detect a difference in tuberculosis cases identified by the two arms.
- We were not able to collect individual data as this would have been very challenging to achieve operationally within the study setting.

## Introduction

Tuberculosis (TB) rates in London are among the highest in Europe, and account for nearly 40% of UK cases.^1^ ^2^ Within London, TB rates are highest for homeless people, prisoners and drug users.^3^ Many of these ‘hard-to-reach’ groups have infectious TB, characterised by delays to diagnosis, poor adherence to treatment and are often lost to follow-up before treatment completion.^3^ Congregate living and reluctance to engage with services complicate screening contacts to identify active cases and latent infections.^4^ Consequently, these patients make a disproportionate impact on control and on the workload of TB services.

Previous research highlighting the problem of TB among hard-to-reach groups led to important service developments including: social workers in specialist TB teams^5^; a project which aims to improve case management of hard-to-reach patients across London and includes mobile digital X-ray unit (MXU) targeted at hard-to-reach groups;^6^ and a national drive to screen all new prisoners in selected prisons using a digital teleradiology network. Qualitative research has shown that peer educators with experience of homelessness and addiction can be beneficial and empowering, and help long-term recovery from TB for that individual.^7^

The pan-London Find and Treat (F&T) TB service includes a MXU and has been shown to be cost-effective among hard-to-reach groups (homeless people, substance misusers and prisoners).^8^ MXUs can identify possible cases of active TB early, often before people become infectious and have the potential to improve clinical outcomes.^9^ ^10^ Historical data from the MXU suggest uptake rates among eligible individuals resident in homeless hostels of around 50%.

Improving the number of individuals screened by the MXU has the potential to increase its cost-effectiveness, reduce the public health risk of infectious TB cases and improve the health of this vulnerable population. Peer educators (individuals with experience of homelessness, problem drug or alcohol use, and TB) are able to have open and honest discussions about sensitive and stigmatised issues such as TB, addiction and engaging with health professionals. Use of peer education has been shown to improve knowledge about health conditions and increase the use of health services in several areas, including HIV, smoking and condom use; however, some of this evidence was based on studies conducted in low income countries and therefore, may not be entirely generalisable to the UK.^11^ ^12^ As a result, peer education is being actively promoted by key organisations (including WHO Europe and UNICEF) in sexual and reproductive health.^13^ Previous qualitative research has found that volunteer peer educators working alongside TB services highly value their involvement and the opportunities it provides for meaningful, structured activity.^7^ However, a recent systematic review of peer education interventions conducted in the European Union found no clear evidence of the effectiveness for HIV prevention, adolescent pregnancy prevention and sexual health promotion for young people.^14^

Using a cluster randomised controlled trial, we aimed to compare current practice in Central London used to encourage homeless people to be screened for TB with the additional use of peer educators, who have direct experience of TB, homelessness or both.

## Methods

The MXU targets high-risk populations in homeless hostels, day centres, drug and alcohol recovery projects, and street populations accessing soup kitchens across London. The study was restricted to hostel populations as these facilities provided a denominator population (number of residents on the day of screening) on which to reliably assess screening uptake. All homeless hostels in London taking part in MXU screening for active pulmonary TB run by F&T (a National Health Service (NHS) led service) were eligible for inclusion in the study if they had taken part in two previous screening sessions. Historical screening uptake data for the previous two rounds were reviewed and those with uptake levels over 80% were excluded from the study. These sites were excluded as it was felt the peer intervention would be unlikely to further improve screening uptake at these locations. Eligible hostels had not been screened in the 6 months prior to the scheduled MXU screening session. There were no additional exclusion criteria. No data about individuals were collected as the intervention was applied at hostel and not at the individual level.

Hostel managers were invited to a meeting hosted by F&T to explain the purpose of the study, and obtain their agreement and consent for participation in the study. Hostel managers unable to attend the meeting were contacted by email and telephoned, where necessary. Hostel managers were reassured that the study was evaluating a peer intervention and not individual hostel performance.

For intervention and control arms, F&T staff were present on the MXU to encourage uptake and manage onward referrals for suspected cases of active TB (usual practice for F&T). At intervention sites, volunteer peer educators arrived at the start of a screening session. Peer educators introduced themselves to the hostel staff and agreed on a work plan. They then moved around the hostel according to the agreed plan of work, knocking on residents’ doors (in conjunction with hostel staff), speaking to residents in all communal areas and those available close to the hostel location (next to the MXU) in order to encourage them to take up the offer of screening.

Peer educator volunteers were recruited via TB clinics in London (and therefore, had experience of TB) or from F&T, where staff identified or were approached directly, by interested service users. Volunteer peer educators, therefore, had experience of TB, homelessness or both. Training for peer educators was provided by attending a 3-day training session run by Groundswell (a registered charity that exists to enable homeless and vulnerable people to take more control of their lives) in conjunction with the research team and F&T, and provided input around TB and what was expected of the peer educators. Training covered issues such as TB transmission, TB risk groups, how treatment is conducted, the importance of screening for active pulmonary disease, how to maximise screening uptake (based on past experiences of F&T staff) and the additional support available for those undergoing screening. Peer educators also underwent a period of shadowing an existing peer educator to learn how to increase screening uptake. Ongoing support for peer educators was provided by Groundswell, based on their experience of working with homeless and vulnerable people to take more control of their lives and to have a greater influence on services they engage with so as to play a larger role in the community.^15^ Additionally, there was quality assurance of peer activity during screening sessions, which covered issues such as time keeping, communication and key peer activities, including how actively they encouraged residents to be screened.

The primary outcome for the study was the number of hostel residents screened for active TB on the MXU at each screening session. The number of residents eligible for screening at each hostel was determined from bed lists at each site, which took account of the number of residents actually staying at a venue overnight and excluded persons who were absent on the day of screening due to hospital admission, arrest or whereabouts unknown.

The study was a cluster randomised trial with a 1:1 allocation ratio between intervention and treatment arms. The unit of randomisation was hostel screening venue. A cluster randomised design was chosen as the intervention was aimed at the hostel sites rather than individual clients. Randomisation was carried out by the study research team using a master list of hostels at the beginning of the study. Sites were randomised to the intervention or control group using the internet-based service by SealedEnvelopeTM (http://www.sealedenvelope.com/), which ensured allocation concealment until interventions were assigned. To ensure comparability between intervention and control arms, hostels were stratified on the basis of their size (binary variable indicating whether hostels had more than 43 beds) and previous screening uptake level (binary variable indicating whether hostels had greater than 50% historical uptake).

On the basis of analysis of historical screening data from F&T, it was estimated that the intracluster correlation coefficient for this study was 0.08. This produced a maximum inflation factor of 6.52, assuming an average hostel size of 70 residents. Many changes to the provision of homeless hostel services took place from the time when the study was conceived to when the recruitment started, including a decrease in the average size of hostels from 70 to 50 residents. As a result, the inflation factor was recalculated as 4.84. To detect a 15% difference in screening uptake in the two groups (60% vs 75%) in an individually randomised controlled trial—with 90% power at the 5% significance level—would require 216 residents in each arm. Applying the 4.84-fold inflation factor for the clustered randomised design required at least 1045 individuals or approximately 21 homeless hostels in each arm.

Blinding of participants and observers was not possible due to the nature of the intervention. The study statistician (RWA) conducted analysis blinded to the allocation of intervention or control arms.

Baseline variable analysis included examining geographical location of the two study sites, hostel characteristics, including bed numbers and occupancy, rating of the hostel performance by research staff using a tool designed for this purpose (see online supplementary appendix), whether incentives were given and the historical levels of screening uptake. The rating of hostel performance included scoring of how involved the manager of the hostel was during the screening session, how much encouragement for screening there was from staff, whether posters advertising the screening were on display, how well-organised the event was and whether incentives were provided on screening day. No individual level data were collected as part of the study; therefore, it was not possible to examine baseline differences between the intervention or control groups, nor to identify any individual who may have crossed over between groups. Missing data were treated as an additional category in all analyses.

Exposure and outcome data at each venue were collected by study nurses present at the time of screening by the MXU on paper data collection forms. These forms were then entered into a Microsoft Excel 2010 spreadsheet by the study database manager. Data were then cleaned by the study statistician and analysed in Stata V.12.

Poisson regression analysis was used to analyse outcome events at screening hostels. Bed occupancy level was included as the exposure variable, screening uptake as the outcome (or indicator) variable, and hostel venue as a random effect to account for clustering at each site. The analysis was adjusted by inclusion of the randomisation stratification factors of historical uptake rates and bed size.

We conducted a secondary per-protocol analysis that only included sites if peer educators turned up for screening sessions and included a binary variable describing whether or not the research staff felt screening had gone as planned. Secondary analysis was also conducted by subgroups, for large and small hostels separately, and for low and high previous screening uptake level using the binary categorical variables described previously for these analyses.

The study trial registration number is ISRCTN17 270 334. The study was registered retrospectively as a result of a series of events. The study team initially believed it would be registered with an International Standard Randomised Controlled Trial Number (ISRCTN) by the National Institute for Health Research as a result of it being entered onto their Clinical Research Network (CLNR) Portfolio Database. The CLNR team informed the study team that the trial did not qualify to be on the portfolio (and as result, would not automatically receive free ISRCTN registration) because it did not involve individual informed consent and therefore, we would not be able to report individual patient recruitment to the CLNR portfolio. The time delay between July 2011 and the start of study recruitment in February 2012 unfortunately contributed to the fact that the lack of trial registration was overlooked by the study team. When RWA began to work on the study and pointed out the trial had not been registered, the CLNR portfolio team were approached for a second time in order to consider the trial for adoption on their portfolio. This request was denied on 14 November 2013 for a second time. The study team, therefore, decided to register the trial independently on ISRCTN and pay for it out of other research funding, but by the time this was arranged recruitment for the study had been completed.

## Results

**Figure 1:**
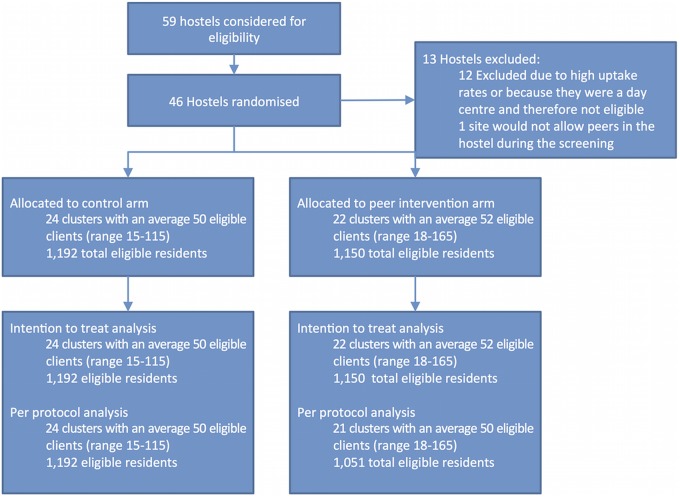
Participant flow diagram.

A total of 59 hostels were considered for eligibility and 46 were included in the study. 12 hostels were excluded due to uptake rates greater than 80% prior to initiation of the study (figure 1). One hostel would not allow peer educators onto the venue during the screening and was, therefore, also excluded. The study took place between February 2012 and October 2013, and at the 46 hostels included in the study, a total of 2342 residents were eligible for screening. A total of nine peer educators took part in the study and recruited residents from a mean of five hostel screening sessions.

**Figure 2:**
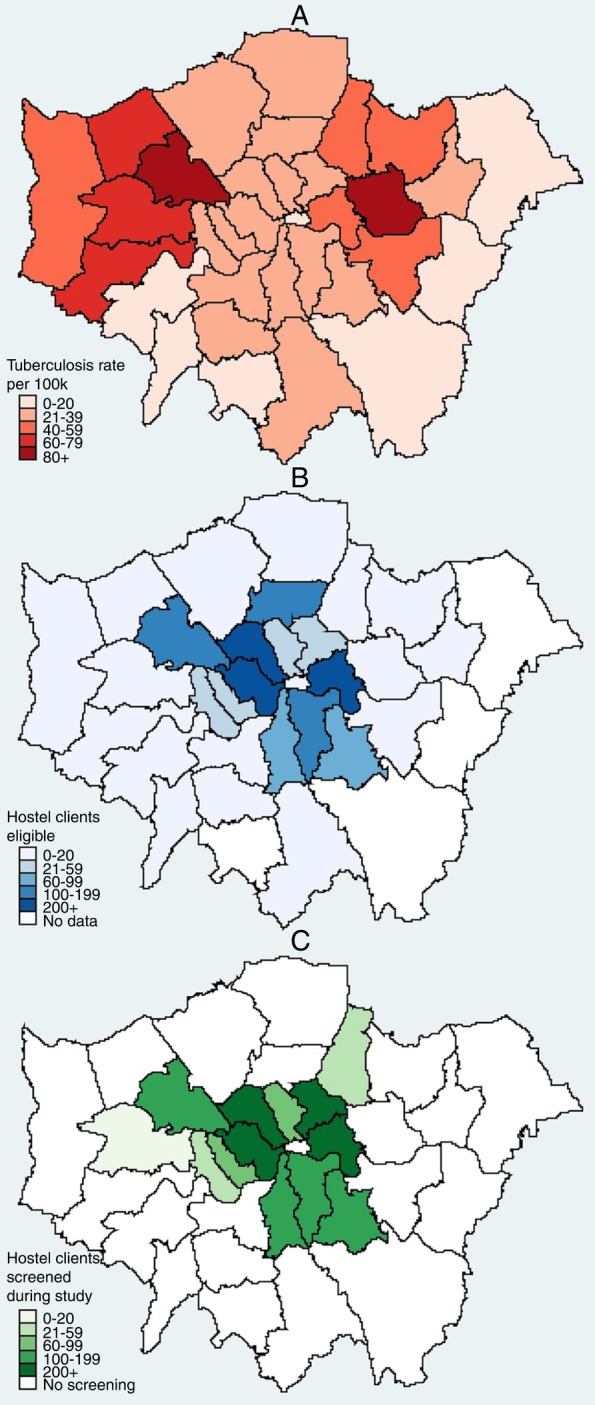
Maps of London by local authority detailing: rates of tuberculosis (A); total eligible clients at all homeless hostels (B); total number of clients screened at hostels as part of the study (C).

There was no evidence of imbalance across any of the baseline characteristics measured (χ^2^ p value >0.47 for all baseline characteristics; table 1). Location of screening sites taking part in the trial was representative of the distribution of homeless hostel places, with the highest levels of eligible clients screened in the London boroughs of Camden, Westminster, Hackney and Tower Hamlets (figure 2).

**Table 1:** Baseline hostel characteristics for intervention and control arms

	Control		Intervention	
	N	(%)	N	(%)
London TB sector*				
North Central	7	29	4	18
North East	6	25	7	32
North West	5	21	8	36
South East	6	25	3	14
Hostel size				
43 or less beds	13	54	12	55
Greater than 43 beds	11	46	10	45
Historical screening uptake				
50% or less	15	63	12	55
Greater than 50%	9	38	10	45
Effectiveness of hostel†				
13 or less	12	50	10	45
Greater than 13	12	50	12	55
Incentives provided for screening‡				
No	15	63	15	68
Yes	9	38	6	27
Unknown	0	0	1	5

*TB control is split into geographical sectors in London.

†Categorisation performed by splitting the data in half; see online supplementary appendix for full details of how this was calculated.

‡May have included food or vouchers for food.

TB, tuberculosis.

Across all sites, median screening uptake was 44% (IQR 26–59). Control sites had a total of 1192 residents, with median uptake of 45% (IQR 33–55). Intervention sites had 1150 eligible residents with median uptake of 40% (IQR 25–61). Using Poisson regression to account for the clustered study design, size of hostel and previous screening uptake, there was no evidence for peer educators increasing uptake of screening with an adjusted risk ratio 0.98 (95% CIs 0.80 to 1.20; table 2).

**Table 2:** Primary analysis of numbers and incident rate ratios for uptake of screening for tuberculosis on the mobile X-ray unit at intervention and control homeless hostels

	Control (N)	Intervention (N)	Total	Unadjusted* intervention group risk ratio (95% CI)	Adjusted† intervention group risk ratio (95% CI)
Number of individuals eligible for screening	1192	1150	2342	–	–
Number of individuals eligible for screening per hostel‡	35 (27, 71)	36 (27, 52)	35 (27, 70)	–	–
Number of individuals screened	503	468	29 (13, 38)	0.96 (0.76 to 1.23)	0.98 (0.79 to 1.21)

*Accounts for clustering at hostel level. †Analysis adjusted for historical uptake rates and hostel bed size and accounts for clustering at hostel level.

‡Data are median (IQR).

Several secondary analyses were conducted as defined a priori. One intervention hostel did not have a peer educator in attendance when the screening took place. A per-protocol analysis found no evidence for peer educators increasing uptake of screening adjusted risk ratio 0.97 (95% CIs 0.78 to 1.22; table 3). No evidence was found for peer educators increasing uptake of screening for any of the other secondary analyses. The study team noted no adverse events.

**Table 3:** Secondary analysis of numbers and incident rate ratios for uptake of screening for tuberculosis on the mobile X-ray unit at intervention and control homeless hostels

	Control Eligible (number screened)	Intervention Eligible (number screened)	Total	Unadjusted intervention group risk ratio (95% CI)	Adjusted* intervention group risk ratio (95% CI)
Per protocol—peer educators who attended intervention hostel on day of screening	1192 (503)	1051 (432)	2243 (935)	0.97 (0.75 to 1.26)	0.97 (0.78 to 1.22)
Hostel that did not participate effectively†	748 (267)	444 (137)	1192 (404)	0.86 (0.67 to 1.11)	0.88 (0.67 to 1.14)
Hostel size					
43 or less beds	362 (176)	338 (134)	700 (310)	0.82 (0.60 to 1.11)	0.80 (0.60 to 1.06)
Greater than 43 beds	830 (327)	812 (334)	1642 (661)	1.04 (0.76 to 1.43)	1.08 (0.82 to 1.42)
Historical screening uptake					
50% or less	694 (272)	718 (241)	1412 (513)	0.86 (0.64 to 1.14)	0.86 (0.65 to 1.14)
Greater than 50%	498 (231)	432 (227)	930 (458)	1.13 (0.85 to 1.51)	1.13 (0.85 to 1.51)

Data are median (IQR) unless otherwise stated.

*Analysis adjusted for historical uptake rates and hostel bed size.

†One hostel (in intervention arm) did not have data collected on participation effectiveness. A score of greater than 13 was considered effective. The rating of hostel performance (results described in table 1) included scoring from 0 to 2 for elements of their effectiveness, including how involved the manager of the hostel was during screening, how much encouragement there was from staff, whether posters advertising the screening session were on display, how well-organised the screening event was and whether incentives were provided on the screening day. See online supplementary appendix for full details of each element included in the hostel effectiveness scoring system.

## Discussion

This cluster randomised controlled trial aimed to compare current practice used to encourage homeless people to be screened for TB, with the additional use of volunteer peer educators who have direct experience of TB, homelessness or both. Using Poisson regression to account for the clustered study design, size of hostel and previous screening uptake, there was no evidence for peer educators increasing uptake of screening. Secondary per-protocol analysis, and restricting data by hostel size and uptake also found no evidence for peer educators increasing uptake of screening.

Our study has high levels of internal validity and used a pragmatic study design in the analysis of a challenging population and setting. The study included most homeless hostels being screened by F&T, a project with broad geographical coverage across London and a focus on areas with greatest need. Particular strengths of the study design include allocation concealment during randomisation and blinded statistical analysis of data by intention-to-treat.

Cluster randomisation was an appropriate design as it would not have been possible to randomise the intervention to individuals. The study design was not powered to detect a difference in TB cases identified by the two arms as this would require considerably larger sample sizes and would have meant repeated screening at hostels, potentially diluting the effect of the intervention during the study. We were not able to collect individual data as part of the study as this would have required individual consent and would have been challenging logistically given that screening took place within an operational setting where any data collection would have interrupted the flow of screening and caused unacceptable delays for service users.

Evidence from systematic reviews of the effectiveness of peer educators’ interventions in different health conditions or behaviours are mixed. One such review looking at a variety of different health conditions or behaviours found evidence for increasing physical activity, decreasing smoking and increasing condom use, but no evidence for breast feeding, medication adherence, women’s health and participation in general activities.^11^ One systematic review of peer-based interventions for HIV also found mixed evidence of effects.^12^ This same systematic review also attempted to examine what implementation factors, including peer educator recruitment, supervision and training, improved the effectiveness of intervention; however, it found a lack of evidence to support any of these particular issues partly as a result of the low sample size.

Several factors may have led to the lack of intervention effectiveness. Most sites where screening took place were not naïve to the peer intervention due to their increased use between the time the study was conceived and when it was conducted. At several sites, in effect we withdrew the ‘intervention’ from the control arm. This may have led to an increased awareness among hostel staff of the techniques employed by the peer educators at the control and intervention sites, reducing any effect during the trial or in fact, preconditioned the sites to the benefits of having peer educators. The primary outcome chosen for the study was screening uptake. This outcome does not take account peer educators previously reported ability^7^ to engage the more difficult to reach and vulnerable cases, which could only be assessed by the collection of individual-level data. Such an effect (if it exists) would result in a reduction of intervention-induced inequalities, and could ultimately lead to an increase in the rate of detection of active TB. Having peer educators available at the time of screening may also help with the engagement of those who are screened and require further healthcare management. This allows for ‘peer advocacy’ with clients, as peer educators are then able to accompany people to follow-up appointments, based on a relationship that was started at the MXU during the peer-education work.

Discussion with both peer educators and staff post-trial raised the issue that while peer educators potentially have greater authenticity with service users, they may lack technical knowledge and confidence to challenge some of the client misconceptions and concerns that reduce screening uptake. It was proposed that the peer intervention element of our intervention may, in fact, be most effective when complemented, that is, delivered in conjunction with professionals. Our study was not designed to test the effectiveness of using peer educators as a stand-alone intervention versus peer educators working alongside professionals, and this remains an important research question.

While the lack of effectiveness implies that this peer-education intervention tested in this study cannot (by definition) be cost-effective, peer educators in this study were associated with minimal costs due to the fact that they were volunteers. Finally, peer educator volunteers themselves may benefit from involvement in structured, meaningful activity as suggested by previous qualitative studies.^7^

We found no evidence for an increased uptake of screening in this study; however, peer educators may have contributed to other unmeasured factors in the screening process. Additionally, involvement of peer educators in the screening is likely to have directly benefited them through training and skills learnt through the research process, factors which were not measured during this study. Further work should attempt to examine whether these ancillary benefits do accrue, including for the peer educators themselves. Qualitative analysis could explore the possible reasons for the lack of effectiveness found by this study, and what aspects of training and delivery make peer education interventions more successful.
